# Applications of solid-state fermented (SSF) diets to improve the growth, organ health, immunity and disease resistance through modulating the transcriptomics and proteomics profile in fish and shrimp

**DOI:** 10.3934/microbiol.2025029

**Published:** 2025-08-05

**Authors:** Shishir Kumar Nandi, Sanchita Sarkar, Md. Toasin Hossain Aunkor, Zulhisyam Abdul Kari, Tanwi Dey, Hien Van Doan, El-Sayed Hemdan Eissa, Mohamad Nor Azra, Muhammad A.B. Siddik, Muhammad Anamul Kabir

**Affiliations:** 1 Department of Aquaculture, Faculty of Fisheries, Sylhet Agricultural University, Sylhet-3100, Bangladesh; 2 Department of Microbial Biotechnology, Faculty of Biotechnology and Genetic Engineering, Sylhet Agricultural University, Sylhet-3100, Bangladesh; 3 Department of Genetic Engineering and Biotechnology, Shahjalal University of Science and Technology, Sylhet, Bangladesh; 4 Department of Agricultural Sciences, Faculty of Agro-Based Industry, Universiti Malaysia Kelantan, Jeli Campus, Jeli 17600, Malaysia; 5 Advanced Livestock and Aquaculture Research Group, Faculty of Agro-Based Industry, Universiti Malaysia Kelantan, Jeli Campus, Jeli 17600, Malaysia; 6 Department of Animal and Aquatic Sciences, Faculty of Agriculture, Chiang Mai University, Chiang Mai 50200, Thailand; 7 Functional Feed Innovation Centre (FuncFeed), Faculty of Agriculture, Chiang Mai University, Chiang Mai 50200, Thailand; 8 Fish Research Centre, Faculty of Environmental Agricultural Sciences, Arish University, El-Arish, Egypt; 9 Institute of Climate Adaptation and Marine Biotechnology (ICAMB), Universiti Malaysia Terengganu (UMT), Kuala Nerus, 21030, Terengganu, Malaysia; 10 Nutrition and Seafood Laboratory (NuSea.Lab), School of Life and Environmental Sciences, Deakin University, Queenscliff, VIC, Australia

**Keywords:** solid-state fermentation, alternative protein, transcriptomics and proteomics, sustainable aquaculture

## Abstract

Fish meal (FM) has long been used as a staple protein source in aquafeed owing to its balanced amino acids, excellent feed conversion, and improved palatability and digestibility. However, the use of FM in aquafeed formulation is facing difficulties due to concerns regarding availability, price, overfishing, and sustainability. Thus, there is a growing interest in seeking alternative protein sources from plant and animal by-products to partially or fully replace FM in aquafeed. Challenges such as lower nutrient bioavailability, high antinutritional factors, indigestible materials, microbial contaminants, and lower palatability issues have limited the incorporation of these protein sources into aquafeed. The application of solid-state fermentation (SSF) strategy represents a sustainable method to address these problems by improving aquafeed quality and introducing health-promoting beneficial microbes. Moreover, numerous studies have shown that SSF enhances growth, feed utilization, health status, immune system, and disease resistance in aquaculture species. At present, molecular approaches such as transcriptomics and proteomics techniques are widely used tools for evaluating the impacts of SSF on fish and shrimp. They provide valuable insights into the mRNA transcripts and proteomes related gene expressions associated with growth, immunity, and stress response. In this article, we outline the requirements for SSF and discussed its role in ameliorating growth, health, immunity, and disease resistance in farmed species. We also provide up-to-date information about the utilization of SSF technology to modulate the transcriptomics and proteomics profiles in fish and shrimp. The complied evidence aims to support future research efforts and encourage the development of fermented feed as a functional dietary option for promoting sustainable aquaculture practices.

## 1. Introduction

Aquaculture occupies a prominent position among the most auspicious sectors in the global food and seafood production industry, playing a pivotal role in satisfying the worldwide need for premium-quality and sustainably sourced protein for consumers. However, the cornerstone for ensuring sustainable aquaculture practices lies in the provision of high-quality fish and shrimp feed [1]. Moreover, feedstuff constitutes the predominant portion in the aquaculture industry, accounting for 60 % of the total production cost [2]. FM has been traditionally used as a major protein source in aquafeed due to its impressive nutritional composition, improved digestibility, and palatability [3]. Despite this, the dwindling supply, exorbitant price, high demand, and subpar quality of FM have limited its application in aquafeed [4],[5]. Additionally, over-reliance on FM contributes to overfishing, raising concerns about the sustainability of marine resources. To mitigate these issues, the use of by-products from the fishing industry, such as enzymes from carcasses and viscera, has emerged as a cost-effective alternative [6]. The recent decrease in FM has led scientists to seek alternative protein sources from non-traditional ingredients, including plants [7]–[12] and animal by-products [1],[13]–[15]. Moreover, wastes or by-products from agriculture and fish processing industries are acknowledged for their multifaceted advantages, such as cost reduction in feed preparation and their role as an environmentally friendly waste management strategy [3],[5],[16]. Non-traditional feed ingredients are locally available at low cost but their direct incorporation into aquafeed is restricted due to the substantial presence of anti-nutritional factors (ANFs) and crude fiber [17],[18]. The solid-state fermentation (SSF) stands out as a highly promising technique that effectively minimizes the presence of harmful substances in these ingredients [19]. Furthermore, SSF can be used in the production of biomolecules of biotechnological interest [20],[21].

SSF involves the cultivation of beneficial microbes such as bacteria, yeast, and filamentous fungi on solid substrates in a controlled environment, playing a critical role in augmenting the nutritional profile of non-traditional ingredients [22],[23]. This innovative approach helps in increasing the bioavailability of essential micronutrients and macronutrients [24],[25]. Furthermore, SSF contributes to enhancing the digestibility and palatability of feedstuffs [3],[26],[27]. The non-traditional fermented feedstuffs are greatly used in the aquafeed for partial or complete replacement of high-value FM, aiming to effectively reduce overall feed expenses [2]. Numerous researchers have observed that substituting FM with SSF protein yields significant benefits in terms of enhancing fish and shrimp growth performance [9],[28],[29], reproduction, and egg quality [17], gut microbiota [19], immune system [22],[30], and disease resistance [31],[32]. In addition, the transcriptomics and proteomics studies serve as valuable tools for evaluating the nutrigenomic aspects of fish and shrimp species. Through comprehensive analyses of gene expression and protein profiles, researchers gain valuable insights into the complex regulatory networks governing physiological processes such as growth, immunity, and stress response. Moreover, the integration of transcriptomic and proteomic data facilitates a holistic understanding of how SSF influences the molecular physiology of aquatic animals. For instance, the fermentation of poultry by-product meal through *Saccharomyces cerevisiae* and *Lactobacillus casei* led to the significant upregulation of the cytokine genes such as IL-8, IL-10, IL-1β, and IL-17F in the gut of *Cherax cainii* [22]. Similarly, the soybean meal (SM) fermented with a mixture of beneficial microbes at levels up to 100% in *Macrobrachium nipponense* diets significantly upregulated the heat shock cognate protein 70 and heat shock protein 90 in the hepatopancreas [33]. Incorporating fermented poultry by-product meal in *O. niloticus* at levels up to 10% significantly upgraded the serum lyz and IgM levels [15]. In this review, we consolidate the knowledge on the application of SSF of feedstuff ingredients on fish and shrimp aquaculture, with a focus on its impact on transcriptomics and proteomics profiles. By synthesizing findings from a diverse array of studies, we aim to provide a comprehensive overview of the molecular mechanisms underlying the beneficial effects of SSF on aquatic animals. Through this study, we hope to inspire further research and innovation in utilizing SSF as a sustainable and efficient strategy to address the growing demand for high-quality fish and shrimp products.

## 2. Requirements for solid-state fermentation

SSF is a microbial process in which bacteria, fungi, or yeasts grow on a solid substrate in the absence or near absence of free water [3]. SSF of feed ingredients offer a sustainable and cost-effective way to enhance the quality of feed, promoting growth and health performance of farmed animals. SSF of feed ingredients necessitate careful consideration of three key elements: Microorganisms, substrates, and favorable environment.

### 2.1. Microorganisms

The choice of microorganisms is crucial in the SSF process. For instance, appropriate microbes can grow and be metabolically active in a water free environment [34], effectively use solid substrates [35], and generate primary and secondary metabolites as well as various enzymes [36]. In general, different types of microbes are applied in the SSF of feedstuffs such as bacteria (*Lactobacillus* spp., *Bacillus* spp., *Enterococcus hirae*, *Astragalus membranaceus*, *Rhodopseudomonas palustris*, and *Bifidobacterium lactis*), yeast (*Saccharomyces* spp.), and fungi (*Aspergillus* spp. and *Rhizopus* spp.) (Tables 1 and 2). Among these, lactic acid bacteria (LAB), often referred to as the powerhouse or key microbes in the fermentation, are widely used throughout the SSF process [26]. The rapid acidification during fermentation due to LAB growth delays the colonization or proliferation of harmful microorganisms in fermented products, thereby prolonging their shelf life [37]. The microorganisms used in SSF can break down complex substances into simpler and more digestible forms. In addition, the application of these microbial organisms in SSF yields various products like lactic acid, acetic acid, or ethanol, with the specific outcome dependent on the species of microbes used [3]. For instance, *Lactobacillus* spp. can generate lactic acid, while yeast can produce ethanol and carbon dioxide [38].

### 2.2. Substrates

In the context of SSF, it is important to take careful consideration of the nature and properties of the substrates used. This involves thoughtful selection and evaluation to ensure that the chosen substrates effectively support the growth of microbes, thereby contributing to the overall success of the fermentation process. Substrates are solid materials, play a crucial role in fermentation, with commonly used varieties including rice straw, wheat straw, rice hulls, corn cobs, and sugarcane bagasse [39]. In SSF, the solid matrix of the substrate absorbs the necessary water content, providing significant advantages for microbial growth and facilitating oxygen transfer [39]. Moreover, in molecular biology, inducers are molecules that regulate gene expression and can either be present in the substrate or added externally. They can act as activators, enabling them to bind to the operator DNA and activate target gene expression. Alternatively, they can act as repressors, binding to the DNA strand to prevent DNA-dependent RNA polymerase from synthesizing mRNA. However, during the fermentation process, it is important to select the right inducer in the microbial media to enhance enzyme production for higher bioprocessing [40]. The lac operon is the most commonly used gene regulatory circuit induced by allolactose, which binds to the repressor and decreases its affinity for the operator site, enabling RNA polymerase to transcribe the operon genes [41]. For instance, Dhillon, et al. [42] tested lactoserum (LAC), derived from lactose substrate obtained from apple pomace, as a crude inducer for fungal enzyme induction in SSF at higher quantities.

### 2.3. Environmental conditions

The environmental conditions such as temperature and pH play pivotal roles, and maintaining their optimal levels is essential for promoting efficient fermentation through the facilitation of microbial growth and the production of enzymes [3],[16],[43]. Different microorganisms and substrates may have specific temperature and pH requirements for optimal fermentation. Moisture is another important factor for microbial activity and the biochemical reactions occurring during SSF. It also affects the physical characteristics of the substrate, ensuring a favorable environment for microbial colonization and growth [44]. Moreover, moisture levels ranging from 60 to 70% are considered suitable for promoting microorganism growth [3]. In addition, during SSF of feed ingredients, the substrate is typically spread in a thin layer with a depth ranging from 1 to 2.5 cm [45]. Furthermore, Zhang, et al. [30] reported that the ideal conditions for the SSF of SM with *B. subtilis natto* are 44 °C for 72 h, 1.0 water to substrate ratio, pH 7.5, and SM layer thickness of 2.0 cm.

**Table 1.** Studies on fermented feedstuff ingredients and their impacts on modulating transcriptomics of aquatic animals.

Aquatic animals	Plant protein sources	Microbes	Duration & inclusion levels	Response in particular dose (organ)	References
**Fish**					
African catfish (*Clarias gariepinus*)	Fermented soy pulp (FSP)	*Lactobacillus* spp.	70 days (0, 25, 50, 75, & 100% FSP)	(↑) TGF-β1, lyzg, and NF-kβ (50% FSP) (Gut & head kidney)	[9]
Hybrid catfish (*Clarias gariepinus* x *C. macrocephalus*)	Fermented vegetable wastes (FVW)	*Enterococcus hirae* UPM02 (0, 2 × 10^5^, 2 × 10^7^ cfu/g)	80, 100, and 120 days	(↑) α2M, CC chemokines, CXC chemokines, LyzC, MYE, and NF-kβ1 p105 (Except control) (Head Kidney, liver, & spleen)	[31]
Largemouth bass (*Micropterus salmoides*)	Fermented soybean meal (FSBM)	LAB, yeast & *Bacillus*	8 weeks (0, 10, 30, & 50% FSBM)	(↓) TGF-β1, IL-10, hepcidin 1 & hepcidin 2 (30 & 50% FSBM) (↑) SOD1 & SOD2 (30% FSBM) (Liver)	[28]
	Fermented Chinese herbal medicine (FCHM)	*L. plantarum*, *B. subtilis*, & *Saccharomyces cerevisiae*	8 weeks (0, 1, 3, & 5% FCHM)	(↑) TGF-β & IL10 (↓) IL8, IL15, & TNF-α (Except control) (Intestine)	[46]
	FCHM	*Clostridium butyricum*, licorice root, *Astragalus membranaceus*, and *Acanthopanax senticosus*	56 days (0, 5, 10, & 20 & FCHM)	(↑) TGF-β, Caspase-3, Tp53 (Spleen) (Except control group)	[29]
Coho salmon *(Oncorhynchus kisutch)*	FSBM	*Sporolactobacillus* spp.	90 days (0, 5, & 10% FSBM)	(↑) TNF-α and IL-6 (0 & 5% FSBM) (Liver)	[47]
Grass carp (*Ctenopharyngodon idella*)	Fermented *Broussonetia papyrifera* (FBP)	*Lactobacillus* spp.	8 weeks (0, 5, 10, 15, & 20% FBP)	(↑) IL-8 &, IL-1β, & Ifn γ (0% FBP) (Intestine)	[48]
Gibel carp (*Carassius auratus gibelio* var. CAS III)	Fermented moringa leaves (FML)	*Aspergillus niger*	50 days (0, 5, 10, & 15% FML)	(↓) TLR2, TNF-α, IL-1β and IL-8 (All diets containing FML) (Spleen)	[30]
Gibel carp (*Carassius auratus gibelio* var. CAS V)	Fermented plant meal (FPM)	*Lactobacillus* spp., yeast & *Bacillus* spp.	60 days (0, 3, 5, & 8% FPM)	(↑) TOR (5 to 8% FPM) (Liver)	[49]
Nile tilapia (*Oreochromis niloticus*)	Fermented olive cake (FOC)	*Aspergillus oryzae*	3 months (0, 5, 10, 15, & 20% FOC)	(↑) TNF-α and IL-1β (10% FOC) (Intestine)	[50]
	Fermented Hilyses	*S. cerevisiae*	60 days (0, 0.2, & 0.4% Hilyses)	(↑) TNF-α and IL-1β (Except 0% Hilyses) (Liver)	[51]
Japanese seabass (*Lateolabrax japonicus*)	SM & FSBM	*B. pumilus* SE5 & *Pseudozyma aphidis* ZR1	8 weeks (0, 40, or 80% SM, BPFSM & PAFSM)	(↑) TNF-α (Except control) (↑) IL-1β (SM80 & BPFSM80 diets) (↓) TGF-β (Except control) (Intestine)	[52]
Hybrid groupers (*Epinephelus fuscoguttatus*♀ × *E. lanceolatus*♂)	Fermented rice protein (FRP)	*Aspergillus oryzae*	56 days (0, 5.98, 17.94, & 29.90% FRP)	(↓) TNF-α, IL-6, & TLR222 (FRP treatments) (Intestinal tract)	[53]
Pearl gentian grouper (*Epinephelus fuscoguttatus*♀ × *E. lanceolatus*♂)	FSBM	*Bacillus* spp.	10 weeks (0, 20, & 40% FSBM)	(↑) IL1β, IL12, IL32, & TNF-α (40% FSBM) (↓) IL5, IL10, IgM, CD4, & Lyz (40% FSBM) (Intestine)	[54]
Crayfish (*Cherax cainii*)	Fermented poultry by-product meal (FPBM)	*Lactobacillus casei* and *S. cerevisiae*	70 days (0 & 70% FPBM)	(↑) IL-8, IL-10, IL-17F (70% FPBM) (Intestine)	[22]
Nile tilapia (*Oreochromis niloticus*)	FPBM	*S. cerevisiae*	8 weeks (0, 10, 20, 30, & 40% FPBM)	(↑) IgM (10 and 20%) (Serum)	[55]
**Shrimp**					
Kuruma shrimp (*Marsupenaeus japonicus*)	Fermented vegetable product (FVP)		11 days (0, 0.55, 5.5, & 55% FVP)	(↔) TLR1 (All treatments) (↑) ALF (5.5, 55% FVP) (Lymphoid organ & intestine)	[56]
*Pacific white shrimp (Litopenaeus vannamei)*	Fermented duckweed flour (*Lemna* sp.) (FDF)	*B. pumilus* and *Pediococcus pentosaceus*	50 days (0, 5, 15, 25, and 35% FDF)	(↔) proPO, TGase, & LvToll (All treatments) (↓) SOD & Lys (0, 25, and 35% FDF) (Hemocytes)	[57]
	FSBM	*S. cerevisiae*, *B. subtilis*, *Rhodopseudomonas palustris*, *& Bifidobacterium lactis*	8 weeks (0, 10, 20, 30, & 40% FSBM)	(↑) Tor, s6k (20% FSBM) (muscle)	[58]
	Fermented rice bran (FRB)	*B. subtilis*	12 weeks (100% basal diet, 50% basal diet + FRB, & only FRB	(↑) Lyz & PO (Except 100% basal diet) (Hemolymph)	[59]
*Macrobrachium nipponense*	FSBM	Mixture of microbes	8 weeks (0, 25, 50, 75, & 100% FSBM)	(↑) CuZnSOD (25% FSBM) (↓) CAT (Except 100% FSBM) (Hepatopencreas)	[33]

**Note:** TGF-β1: Transforming growth factor-β1, Lyz: Lysozyme, α2M: Alpha-2 macroglobulin, MYE: Myeloperoxidase, TNF-α: Tumor necrosis factor alpha, Tp53: Tumor protein-53, IL: Interleukin, CC-chemokines: Cysteine-Cysteine chemokines, CXC chemokines: Cysteine-X-Cysteine chemokines, LyzC: Lysozyme-C, NF-kβ1: NF-kappa-β1, Ifn-*γ*: Interferon-*γ*, NF-β: Nuclear factor-β, TLR: Toll-like receptor, TOR: Target of rapamycin, IgM: Immunoglobulin, ALF: Anti-lipopolysaccharide factor, SOD: Superoxide dismutase, CAT: Catalase, proPO: prophenoloxidase, TGase: Transglutaminase, LvToll: Toll receptor, PO: Phenol oxidase, and s6k: s6-kinase. **Signs:** (↑) indicate up-regulated genes, (↓) indicate down-regulated genes, and (↔) indicate no effects on gene expression.

**Table 2.** Studies on fermented feedstuff ingredients and their impacts on modulating proteomics profile of aquatic animals.

Aquatic animals	Plant protein sources	Microbes	Duration & inclusion levels	Response in particular dose (organ)	References
**Fish**					
Pearl gentian grouper (*Epinephelus fuscoguttatus*♀ × *E. lanceolatus*♂)	FSBM	*Bacillus* spp.	10 weeks (0, 20, & 40% FSBM)	(↑) Aqu8, Aqu9, Aqu10, jam, claudin3, claudin12, claudin15, and ZO-3 (40% FSBM) (↓) Aqu1, Aqu4, and Aqu12 (40% FSBM) (↔) Aqu11, ZO-2, guanylin, nkaα-1, nkaβ-1, and nkaγ (All treatments) (Gut)	[54]
Grass carp (*Ctenopharyngodon idella*)	FBP	*Lactobacillus* spp.	8 weeks (0, 5, 10, 15, & 20% FBP)	(↑) Keap1 (20% FBP) (Intestine)	[48]
Golden pompano (*Trachinotus ovatus*)	Fermented cotton seed meal (FCSM)	*Bacillus* spp.	56 days 0, 12.5, 25, 50, & 100% FCSM	(↑) ZO-1, claudin-3 and claudin-15 (25% FCSM)	[60]
Japanese seabass (*Lateolabrax japonicus*)	SM & FSBM	*B. pumilus* SE5 & *Pseudozyma aphidis* ZR1	8 weeks (0, 40, or 80% SM, BPFSM & PAFSM)	(↑) HSP70 (All dietary groups of SM and PAFSM) (Intestine)	[52]
African catfish (*Clarias gariepinus*)	FSP	*Lactobacillus* spp.	70 days (0, 25, 50, 75, & 100% FSP)	(↑) HSP90a (50 and 75% FSP) (Gut & head kidney)	[9]
Turbot (*Scophthalmus maximus* L.)	FSBM	*Saccharomycopsis fibuligera* Y27, (SF) *B. tequilensis* KCTC 13622, (BT) *B. subtilis* D7XPN1, (BS) and *B. aryabhattai B8W22* (BA)	10 weeks (Control, SF45, BT45, BS45, and BA45)	(↑) *Agrp* (SF45) (↓) *Pomc* (SF45) (Gut)	[61]
	SM and FSBM	*Shewanella* spp. MR-7	79 days (0, 15, 30, 45, & 60% SM and FSBM)	(↓) Occludin, *ZO-1*, and tricellulin (≥30, ≥45, & ≥45% SM respectively and 60% FSBM) (Gut)	[62]
Largemouth bass (*Micropterus salmoides*)	FCHM	*Clostridium butyricum*, licorice root, *Astragalus membranaceus*, and *Acanthopanax senticosus*	56 days (0, 5, 10, & 20 & FCHM)	(↑) ZO1 and Claudin-4 (Intestine) (Except control group)	[29]
Hybrid catfish (*Clarias gariepinus* x *C. macrocephalus*)	FVW	*Enterococcus hirae* UPM02 (0, 2 × 10^5^, & 2 × 10^7^ cfu/g)	80, 100, and 120 days	(↑) BPIP (Except control) (↓) HSP70 (Except control) (Head Kidney, liver, & spleen)	[31]
**Shrimp**					
*Macrobrachium nipponense*	FSBM	Mixture of microbes	8 weeks (0, 25, 50, 75, & 100% FSBM)	(↑) HSC70 (0 & 100% FSBM) (↑) HSP90 (100% FSBM) (Hepatopencreas)	[33]

**Note:** Aqu: Aquaporin, Jam: Junctional adhesion molecule, Nkaα-1: Na+/K+-ATPase alpha-1, Nkaβ-1: Na+/K+-ATPase bita-1, and Nkaγ: Na+/K+-ATPase gamma, Keap1: Kelch-like ECH-associated protein 1, Agrp: Agouti gene-related protein, Pomc: Proopiomelanocorein, ZO: Zonula accluden, HSP: Heat shock protein, BPIP: Bactericidal permeability-increasing protein, and HSC70: Heat shock cognate protein-70. **Signs:** (↑) indicate up-regulated genes, (↓) indicate down-regulated genes, and (↔) indicate no effects on gene expression.

## 3. Role of solid-state fermented ingredients in modulating growth, immune response, and disease resistance of fish and shrimp

### 3.1. Role of solid-state fermented ingredients in modulating the growth performance of fish and shrimp

Several researchers have examined the possible impacts of solid-state fermented ingredients on the growth performance of aquatic animals, especially in fish and shrimp. The fermentation process plays a key role in breaking down the complex protein into more digestible protein and free amino acids, which subsequently results in enhanced growth performance due to increased digestion and absorption of diet nutrients. Shrimp, *Fenneropenaeus indicus*, were fed various levels of solid-state FSBM (0, 25, 50, 75, and 100%) for 90 days of the experiment, and the results revealed that FM replaced with FSBM at approximately 50% did not compromise the final weight, weight gain, specific growth rate, and survival of that species [63]. Rui, *L. rohita*, diets fermented with *S. cerevisiae* showed remarkable enhancement in fish overall growth performance when compared to a non-fermented control diet [64]. A 49-day experiment on Nile tilapia, *O. niloticus*, fed with commercially available SSF products notably modulated the overall growth and feed utilization performances, characterized by enhanced weight gain, specific growth rate, feed conversion, protein conversion, and condition factor [65]. Moreover, plant-derived feed fermented with *L. acidophilus* significantly improved the survival rate and feed intake in Nile tilapia, *O. niloticus* [66]. Common carp, *C. carpio*, diets formulated with incorporation of 0.1% SSF supplement positively influenced the growth and feed utilization performance [67]. Dietary rice bran and wheat bran fermented with *B. subtilis* and *B. coagulants* had positive consequences on the overall growth performance in terms of enhanced weight gain, FCR, and PER in *C. carpio* L. [68]. The final weight, weight gain, SGR, FCR, and PER in Nile tilapia were significantly increased when diets were formulated with fermented wheat bran with an inclusion level up to 20% [69]. Novriadi, et al. [70] evaluated the potential impacts of replacing SM with FSBM at 0, 25, 50, 75, and 100% levels in Florida pompano (*Trachinotus carolinus*) growth performance. The outcomes of this study demonstrated no adverse effects on the growth indices of fish such as final weight, percent weight gain, FCR, and thermal unit growth coefficient among the dietary treatments. *D. labrax* fed a diet containing 0, 10, and 20% SSF of Brewer's spent grain modulated growth-related parameters such as final weight, weight gain, daily growth index, feed intake, and PER [71]. These outcomes collectively indicate that SSF can significantly promote the growth performance of aquatic species, likely through enhanced nutrient bioavailability, reduced ANFs, and enrichment with beneficial microbial metabolites.

### 3.2. Role of solid-state fermented ingredients in improving intestinal and liver health of fish and shrimp

The intestine and liver are two vital accessory organs for evaluating the digestion and absorption of dietary nutrients in fish and shrimp. Numerous studies have reported that the SSF strategy significantly modulates the gut and hepatic health in aquatic animals by enhancing the nutrients' bioavailability and digestibility. For instance, Anwar, et al. [67] reported that *C. carpio* diets treated with the SSF supplement upgraded the intestinal architecture, characterized by higher goblet cells and wider lamina propria as well as a villi structure compared to the control group. *C. carpio* L. that received *Bacillus* fermented rice and wheat bran remarkably enhanced the gut mucosal integrity compared with the non-fermented treatment groups [68]. Mohammady, et al. [69] recorded that *O. niloticus* fed diets containing different degrees of *S. cerevisiae* fermented wheat bran (0, 15, 20, and 25%) for 70 days, and their results indicated that up to 20% inclusion of fermented wheat bran in the fish diet enhanced the gut morphology. Zhang, et al. [54] also reported that Pearl gentian grouper diets formulated with 20% and 40% FSBM notably reduced the gut plica length and width and microvilli length, as well as increased the lamina propria width in fish as compared to the control fish. Another study noted that *O. niloticus* fed diets having 0, 25, and 50% FSBM demonstrated substantial improvement in gut architecture, including enhanced goblet cells and gut villi integrity, while these benefits diminished at higher inclusion levels of 75 and 100% [72]. Wang, et al. [73] evaluated the effects of partial replacement of SM with *Monascus purpureus* M-32 FBSM (0, 20, 40, and 60% MFSM) in *L. vannamei*. This study exhibited that villi height was greatly enhanced with the rise of MFSM inclusion, and the intestinal myenteric thickness was substantially higher in all experimental groups compared to the control.

Zebrafish *D. rerio* treated with 1% SSF product of *B. subtilis* HGcc-1 reduced the adverse impacts on the liver by lowering the hepatic lipid accumulation [74]. Liver health indicators such as nuclear change, glycogen granulation, and inflammation were found in a better state when dietary SM replacement increased with the increase of FSBM in *T. carolinus* diets [70]. According to Zakaria, et al. [75], substituting FM with FSBM at levels up to 40% demonstrated considerable enhancement in the numbers of sinusoidal cytoplasm, nuclei, and erythrocytes, and a decrease of vacuolar cytoplasm in the liver of *C. gariepinus*. This study also revealed that FM replacement with FSBM at higher inclusion levels (60 and 70%) had adverse impacts on liver health. Siddik, et al. [76] noted that dietary FM replaced with non-fermented and fermented tuna hydrolysate at levels of 0, 50, and 75% in Barramundi *Lates calcarifer* had shown significant alterations in the hepatic health and maintained their optimum health up to 50% tuna hydrolysate (fermented and non-fermented) inclusion. However, 75% tuna hydrolysate incorporation showed adverse impacts on liver structure by increasing vacuolation, fat deposition, and necrosis. In *O. niloticus*, dietary inclusion of FSBM at 0, 25, and 50% maintained normal liver morphology, whereas higher levels (75 and 100%) led to increased vacuolation, hepatocyte hypertrophy, and necrosis [77]. In *C. carpio*, graded levels of SSF products of yeast (2, 3, 4, and 5 g/kg SFPY) substantially reduced the vacuolar cytoplasm and infiltration of inflammatory cells in the fish hepatic tissue in comparison with the control fish (0 g/kg SFPY) [78]. Overall, SSF feed ingredients greatly increased the intestinal and liver health in fish and shrimp by lowering the ANFs, increasing nutrient digestibility, and providing probiotics, prebiotics, and bioactive substances that boost immunity, modulate gut microflora, and decrease oxidative stress.

### 3.3. Role of solid-state fermented ingredients in enhancing immune response and disease resistance of fish and shrimp

The application of SSF in aquaculture diets has received considerable attention because of its ability to modulate the immune system in various fish and shrimp. Recent studies have confirmed that SSF ingredients can act as natural immunostimulants, increasing both innate and adaptive immune systems in aquatic organisms. For example, Das, et al. [64] reported that non-specific immune systems, including hemolytic activities, digestive enzyme activities, and lysozyme, as well as myeloperoxidase activities in *L. rohita* were significantly upgraded when fed fermented diets as compared to those fed with non-fermented diets (control). A four-week experiment on *D. rerio* fed with 1% SSF product of *B. subtilis* HGcc-1 significantly elevated the epidermal mucus immune system in fish [74]. *S. cerevisiae* fermented wheat bran incorporated in *O. niloticus* diets as high as 20% substantially enhanced the hematological indices such as white blood cells, lymphocytes, monocytes, neutrophils, hematocrit, hemoglobin, mean corpuscle hemoglobin, and mean corpuscle hemoglobin concentration, and decreased the serum biochemical parameters, including LDL, HDL, VLDL-cholesterol, and triglycerides [69]. SM replaced with FSBM at 0, 25, 50, 75, and 100% levels in *T. carolinus* showed no remarkable alterations in the serum albumin, total protein, alkaline phosphatase, cholesterol, glucose, alanine aminotransferase, and aspartate aminotransferase contents in fish [70]. European seabass *Dicentrarchus labrax* receiving SSF of *Gelidium corneum* by-product treated diets revealed no adverse effects on the peroxidase and lysozyme activities [79]. FM replaced with FSBM at levels up to 20% significantly enhanced the immunological parameters, such as serum total protein and lysozyme activity in *L. vannamei* in comparison with other treatment and control diets [80]. The inclusion of FSBM as high as 40% in African catfish *C. gariepinus* diets as FM and SM replacers notably elevated the white blood cell, lymphocyte, red blood cell, hemoglobin, and hematocrit contents in comparison with other FSBM levels [75]. The increased immune mechanism observed in the above-mentioned studies might be due to higher nutrient bioavailability and the dominance of beneficial microbiota in the SSF feedstuffs ingredients.

SSF ingredients in aquafeeds have also been shown to improve the disease resistance in fish and shrimp by modulating the intestinal microflora. Achmad, et al. [81] documented that Marron *Cherax cainii* treated with *S. cerevisiae* and *L. casei* fermented abalone waste (FMAS) at 0, 25, 50, 75, and 100% for 90 days. The result of this observation revealed that different levels of FMAS that replaced FM significantly enhanced the percentage of survival (100%) in fish when challenged with *Vibrio mimicus* for 96 hours. Moreover, Wang, et al. [78] examined the impacts of graded levels of SFPY on disease resistance in *C. carpio*, and their result displayed that SFPY supplementation significantly boosted the resistance to spring viremia carp virus in fish. The partial substitution of SM with MFSM discovered notable enhancements in the disease resistance in *L. vannamei* upon being challenged with *V. parahaemolyticus* [73]. The inclusion of *Bacillus* FSBM as a partial substitution of FM significantly decreased the cumulative percentage mortality (CPM) in *L. vannamei* after being challenged with *V. parahaemolyticus* [82]. Overall, the findings of these reports suggested that incorporating SSF ingredients into aquafeeds denotes a sustainable and functional approach to boost the immune mechanism in different fish and shrimp and increase resistance to pathogenic infestation. Nevertheless, future research is required to optimize fermentation conditions, bacterial strains, and substrate combinations to maximize immunomodulatory benefits across aquaculture species.

## 4. Role of solid-state fermented ingredients in modulating transcriptomics and proteomics profile in fish and shrimp

**Figure 1.**
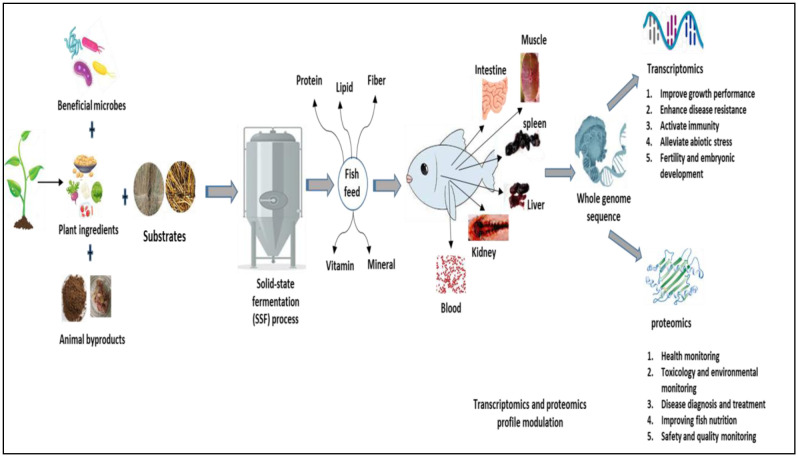
An overview of the application of the SSF strategy in modulating transcriptomics and proteomics profiles in Aquaculture.

Tables 1 and 2 summarize the impacts of SSF of feedstuff ingredients in stimulating the transcriptomics and proteomics profiles in fish and shrimp. The modulation observed in aquatic animals is attributed to the role of the SSF process in influencing nutrient composition, providing bioactive compounds, modulating intestinal microbiota, and regulating immune function (Figure 1). Understanding these interactions is essential for optimizing the use of fermented ingredients in aquafeed formulation to enhance the growth, health, and sustainability of aquatic animals. Juvenile Turbot fed diets with varying proportions of FSBM for 79 days showed significant differences in the expression of intestinal inflammatory genes, with fish receiving 60% FSBM displaying elevated IL-1β and TNF-α levels, while TGF-β1 levels remained stable [62]. Additionally, replacing FM with FSBM at concentrations of 0, 5, 10, and 15% notably altered the expression of protein metabolism-related genes in the hepatopancreas of Chinese mitten crab. Genes such as protein kinase-B, S6 kinase-1, oligopeptide transporter-1, and 4E-binding protein-1 showed significant upregulation compared to the control diet [83]. In an eight-week study, replacing FM with FSBM at 100% in *L. vannamei* diets led to the upregulation of genes like igbp, traf6, and hsp70 genes, when compared to the other treatment groups [84]. Moreover, comparable beneficial impacts of FSBM have been found in other aquatic species, including *Lateolabrax japonicus* [52], *Litopenaeus vannamei* [58], and *Oncorhynchus kisutch* [47], as noted in Table 1.

In *H. molitrix*, incorporating FRB at a level up to 20% in experimental diets revealed significant upregulation of enzymatic genes such as SOD, CAT, and GSH-Px contents [85]. Omarini, et al. [86] stated that rice bran serves as a superb reservoir of protein, amino acids, fatty acids, and antioxidant compounds. Feeding juvenile Gibel carp with FML resulted in a significant reduction in spleen immune-related gene expression (TLR2, IL-8, IL-1β, and TNF-α) after an *Aeromonas hydrophila* challenge compared to pre-challenge levels [30]. Likewise, the incorporation of different levels of moringa leaves (0.5, 1, and 2%) into crayfish *Procambarus clarkii* diets greatly affected the stress-related antioxidant genes such as SOD, CAT, MDA, and GSH-Px [77]. The presence of essential nutrients, beta-carotene, phytochemicals, and natural antioxidants in FML may contribute to improved health and strengthened immune systems in aquatic animals.

*L. vannamei* were provided with FDF for 50 days showed significantly higher expression levels of cathepsin-B, HSP70, and HSP90 with 15%, 25%, and 35% FDF, and elevated lyz levels with 5% and 15% FDF compared to the control [57]. The fermentation process can enrich the nutritional value of duckweed by reducing the crude fiber and ANFs. This improvement potentially stimulates the mRNA transcript and protein-related genes in fish and shrimp. Incorporating FCSM at levels of 0%, 12.5%, 25%, 50%, and 100% into the diets of Golden pompano significantly affected immune-related gene expressions. Notably, IL-10 expression increased in the 12.5% FCSM group, while IL-1β and TNF-α were upregulated in the 12.5%, 50%, and 100% FCSM groups. Fish on a 100% FCSM diet showed significantly increased IL-8 and NF-kβ levels, and the 25% FCSM group had upregulation of ZO-1, Claudin-3, and Claudin-15 [60]. Microbial fermentation enhances the extraction of phenols from cottonseed meal, increasing its protein content and producing small peptides and growth-modulating factors that enrich its nutritional value [60] and potentially activate molecular mechanisms in aquatic organisms.

The inclusion of 70% FPBM in the diets of freshwater crayfish resulted in significant upregulation of immune-related genes such as IL-8, IL-10, and IL-17F in the gut compared to the basal diet [22]. Fermentation breaks down proteins in poultry by-products meal into small peptides and free amino acids, making them easily digestible for fish and shrimp, which enhances gut microbial load and significantly improves gut health, enzymatic activities, and transcriptomic and proteomic profile.

Other non-traditional fermented feed ingredients such as vegetable wastes, sweet potato residue, Chinese herbal medicine, olive cake, and Hilyses have also been used in animal feeds to explore their effects on gene expression and protein expression in fish and shrimp. For example, Elshopakey, et al. [56] found that feeding *Marsupenaeus japonicus* varying amounts of FVP resulted in significant enhancements in anti-lipopolysaccharide factor, although there were no observable effects on the TLR-1 gene. Fermented vegetable wastes/products contain a complex mixture of microorganisms, nutrients, antioxidants, and antimicrobial and immunomodulatory agents that contribute to their potential effects on gene expression, immune function, and health in aquatic species. *L. vannamei* consumed fermented sweet potato (FSP) residue at approximately 28% exhibited significant upregulation of proteome genes associated with the mTOR pathway, including v-type proton ATPase subunit G/F and ribosomal protein s6 kinase [87]. Similarly, FM replaced with FSP up to 28% revealed significant enhancement of Lyz and peroxidase (POD) enzymatic activities in *L. vannamei*. However, Lyz, SOD, and POD activities were greatly reduced in higher inclusion of FSP (86%) [88]. Fermentation might bolster the bioavailability and bioactivity of nutrients in FSP, leading to possible impacts on stress and proteome-related gene expressions. Graded levels of FCHM resulted in a notable rise in TGF-β and IL-10 genes and a decrease in IL-8, IL-15, and TNF-α genes in *M. salmoides* [46]. Maturana, et al. [89] reported yeast biomass in fermented Hilyses as rich in essential nutrients, with cell wall components like β-glucans and mannans exhibiting immunomodulatory properties. Nile tilapia fed diets supplemented with 0%, 5%, 10%, 15%, and 20% *Aspergillus oryzae* FOC for 90 days showed a significant increase in TNF-α and IL-1β gene expression in the gut for the 10% FOC group compared to other treatments [50]. Furthermore, olive cake contains phenolic compounds such as tyrosol, oleuropein, and hydroxytyrosol, which have anti-oxidant, anti-bacterial, and anti-inflammatory properties [90]. Fermentation might augment the bioavailability and functionality of these bioactive compounds and modulate immune response and gene expression related to inflammation and immune function in fish.

## 5. Conclusions

In summary, the application of the SSF strategy in modulating growth, organ health, immunity, disease resistance, transcriptomics, and proteomics profiles in fish and shrimp presents a promising avenue for advancing the aquafeed industry. Through an extensive review of recent works, it becomes evident that SSF offers unique benefits in increasing nutritional quality, bioactive compound production, flavor, and safety of aquaculture products. By harnessing the microbial diversity and enzymatic activities inherent in SSF, researchers have been able to explicate the complex gene expression patterns and protein synthesis pathways, leading to a deeper understanding of how SSF can positively influence farmed animals. Moreover, SSF demonstrates great possibility for sustainable aquaculture practices, offering environmentally friendly solutions to challenges by increasing growth, nutrient utilization, immunity, disease resistance, and overall health of aquatic organisms.

## Use of AI tools declaration

The authors declare they have not used Artificial Intelligence (AI) tools in the creation of this article.
